# Experimenting Sensors Network for Innovative Optimal Control of Car Suspensions

**DOI:** 10.3390/s19143062

**Published:** 2019-07-11

**Authors:** Gianluca Pepe, Nicola Roveri, Antonio Carcaterra

**Affiliations:** Department of Mechanical and Aerospace Engineering, Sapienza University of Rome, 00184 Rome, Italy

**Keywords:** semi-active suspension, control, sensors network, car vibration

## Abstract

This paper presents an innovative electronically controlled suspension system installed on a real car and used as a test bench. The proposed setup relies on a sensor network that acquires a large real-time dataset collecting the car vibrations and the car trim and, through a new controller based on a recently proposed theory developed by the authors, makes use of adjustable semi-active magneto-rheological dampers. A BMW series 1 is equipped with such an integrated sensors-controller-actuators device and an extensive test campaign, in real driving conditions, is carried out to evaluate its performance. Thanks to its strategy, the new plant enhances, at once, both comfort and drivability of the car, as field experiments show. A benchmark analysis is performed, comparing the performance of the new control system with the ones of traditional semi-active suspensions, such as skyhook devices: the comparison shows very good results for the proposed solution.

## 1. Introducing Key Performance Indexes (KPI) for Suspension Systems

Semi-active suspension systems are part of the natural mechatronic evolution of cars. However, many contributions come from other suspension-related field. In fact, their use presents advantages in several different fields ranging from motor vehicle suspension control [1,2,3], to rail vehicles [4], from airplane landing [5], to marine vehicles [6,7], from vibration control of structures [8,9], and in general to any shock absorber process [10]. Comfort is a primary goal in that controllable damping makes more effective the suspension filtering of the road roughness. It is known that noise and vibrations on board can be substantially reduced by acting on the suspension parameters, such as damping and stiffness. At the same time, when making better comfort performance, something is lost in terms of handling. Softening the suspension has, in fact, potentially negative effects on the tire-road contact forces and on car rolling response. An optimal balance of these opposite performance requirements, needs deep analysis and definition of suitable key performance indicators, as discussed ahead.

This paper considers the solution to the previous technical problem through a semi-active technology, installing on board of a real car, a new integrated sensors-controller-actuators device. The system acquires information on the car response through the deployment of a network of sensors, that produces in this way a primary source of data. These are elaborated by an electronic controller that is based on a very new control algorithm, variational feedback control (VFC), recently proposed theoretically by the authors, and for the first time experimentally implemented as illustrated in the present paper. The controller drives a set of semi-active actuators of magneto-rheological type [11,12,13].

Semi-active control based on VFC was firstly reviewed in [8,14,15,16,17] and was introduced in order to increase handling and comfort performance in car suspension. Their control logics were mainly derived by numerical simulations, which optimized some key parameters related to comfort and handling, in order to reduce the acceleration of the car body and increase the contact force exchanged from the tire and the road. In the light of these results, it is the authors’ opinion that VFC control logic is able to provide an excellent tradeoff between antithetical requirements for car dynamics, such as having a comfortable car that is also characterized by a sharp handling: these are almost unique features of the VFC, in respect to others’ control logic [18,19,20].

Concerning the design of a new suspension, its final performances are influenced by several parameters: as an example, in order to maximize comfort, the goal is to limit vertical accelerations transmitted to the car seats, while to enhance the drivability, the tire adhesion to the road should be maximized by the suspension system, guaranteeing, as much as possible, a constant load on the contact patch, minimizing load drops due to road irregularities. In this regard, the suspension stroke is one of the most important parameters to design: the car body acceleration can be indeed easily controlled if large movements of the car body, i.e., of the elastic elements of the suspension, are allowed. By contrast, if the movements of the car body are strictly constrained, for instance by exterior design requirements (limited space requirement from tire and fender), limiting the vertical acceleration becomes a task much more difficult to obtain. Another important parameter of the suspension setup that deserves to be properly designed is the settling time, which however is not explicitly linked to the handling or comfort performances. 

In order to deliver a general optimal control of the suspension system, a number of key performance indexes (KPI) are here introduced. Considering pairs of these KPI to be associated with the x and y-axes, they can define special performance planes, where some special regions can be linked to the different existing suspension’s control logic. In this regard, the performance of a new control method can be evaluated by the capability of reaching zones not accessible to the conventional controllers. The introduction of VFC theory and its application to car dynamics is here presented, highlighting how the control law is able to sneak into problematic regions of the performance planes because they are characterized by different and antithetic features of the suspension. 

The VFC control logic is suitably tuned through numerical models that emulate both the behavior of the car and the response of variable damping actuators. In the sections below it will be shown how the VFC is carefully tuned with nonlinear dynamic models of half-car, rather than the more usual quarter-car model. This choice is motivated by the fact that the vehicle wants to be studied by observing both, its behavior during longitudinal motion on long road routes and how it behaves during the crossing of bumps or holes. The coupled heave and pitch dynamics allow indeed a more realistic check of the performance of the controlled response and, therefore, permits an in-depth study of the performance of the proposed suspension system. The suspension is indeed required not only to avoid relevant accelerations at the point that is attached to the car body, a task that can be carried out, for example, considering a simpler quarter-car model, but also the quality of control should be evaluated monitoring the motion of the passengers seats, that is influenced by pitch and heave motion of the car body and that is driven by the suspension system of the half-car. In addition, several abilities of the suspension should be taken into account to be confronted with different kinds of excitations: one kind of excitation is the random force induced by a rough road and the capability to filter this kind of excitation is surely a good indicator to classify the job done by the suspension, however this capability of the suspension is also confronted with the bump response, which generally is obtained acting on a completely different type of setting. 

In this regard, a number of different parameters (KPI) are identified to be monitored for analyzing the comfort and handling of the vehicle, such as the settling time and maximum acceleration after a speed bump crossing or rather the mean vertical body acceleration and wheel displacement on extra-urban driving cycle. The goal of this publication is indeed the application of the VFC to a real car, i.e., the BMW 1 series, where all the KPI are evaluated. The KPI analyzed are grouped into two categories: time step-response and statistical analysis of which only the most relevant are listed ahead. 

A crossing bump simulation is employed to calculate to the former set of indexes:

- $J_{peak}=\max\left(\left|{\ddot{z}}_{b}\right|\right)$ the largest vertical body acceleration ${\ddot{z}}_{b}$ reached by the vehicle;
- $J_{hand}=\sigma_{\Delta }$ the variance of the stroke Δ reached by the front and rear suspension, representing the analysis index of the handling of the vehicle;
- $J_{settling}=t_{settling}:\;\left|\Delta \right|<\varepsilon$ the settling time required to the suspension for dissipating the involved mechanical energy, which brings the oscillation of the suspension below the threshold value *ε*.

The second set of indexes are evaluated for extra-urban driving cycle:

- $J_{comf}=\sigma_{{\ddot{z}}_{b}}$ the variance of the vertical body acceleration defines the comfort index;
- $J_{hand}=\sigma_{\Delta }$ the variance of the stroke Δ reached by the front and rear suspension, representing the analysis index of the handling of the vehicle;

KPI evaluated in the frequency domain are typically filtered within a suitable band-pass, for instance, the KPI related to comfort $J_{comf}$ is generally analyzed between 0–20 Hz, while the KPI related to handling and $J_{hand}$ is analyzed between 0–30 Hz. 

**Figure 1:**
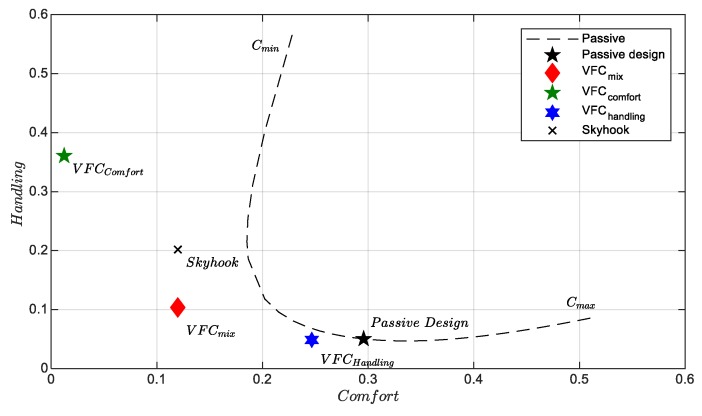
Handling vs. comfort index in the extra-urban cycle.

**Figure 2:**
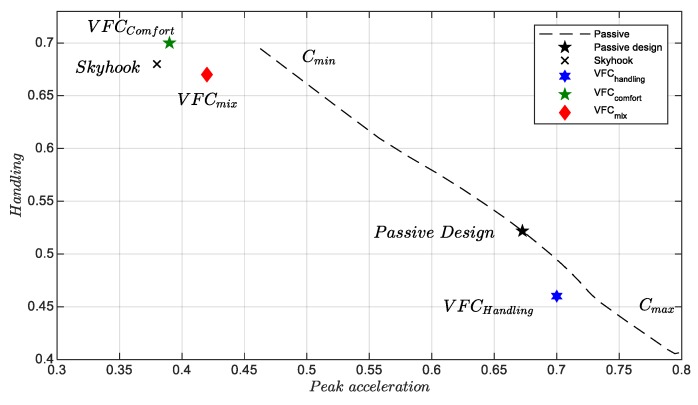
Maximum peak acceleration vs handling, over a speed bump.

**Figure 3:**
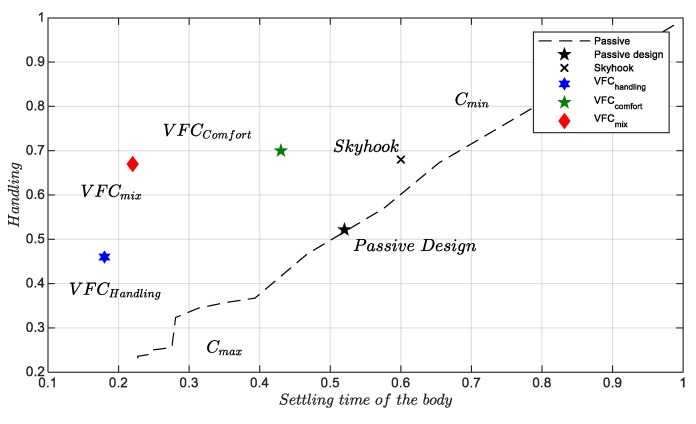
Body settling time vs. handling, over a speed bump.

The Figure 1, Figure 2 and Figure 3 show some pairs of KPI computed via experimental test: five different simulation benchmarks are analyzed, i.e., the original passive suspension vs, four different control strategies, the Skyhook [21], and three VFC tuning [14]. Furthermore, in all planes, there is a dashed curve which represents the technological limit of a passive damper in which the damping factor is constant. This curve was found through a rough interpolation of experimental tests in which the damping coefficient was kept constant between a minimum damping factor $C_{min}$ and the maximum $C_{max}$. In this respect, the passive and Skyhook suspensions are generally used as a reference in order to benchmark the performances of other semi-active controls. The indexes-plane is organized to compare two J indexes at once: in each plan, the markers represent the index values reached by each considered control law or by the passive suspension setting, the origin of the axes represents the optimal minimum J. All the quality evaluation diagrams of the different tests in comparison are normalized with appropriate coefficients to present the results at best. In the forthcoming chapter, it is explained how the proposed control logic is fine-tuned to obtain the best KPI explaining the hardware and sensor used and the experimental campaign conducted. 

Figure 1 shows the plane for assessing comfort and handling for an extra-urban route, i.e., the $J_{comf}$ and $J_{hand}$ plane (the smaller the index, the better the performance). Three different VFC control tunings are compared. The first one called $VFC_{comfort}$ which is tuned specifically to improve comfort minimizing the vertical accelerations, then we have $VFC_{handling}$ which aims to improve road holding and finally an intermediate performance between the previous two, $VFC_{mix}$. These results concern experimental tests on extra-urban routes with average travel speeds between 70–110 km/h. The skyhook has excellent results in optimizing comfort even if the $VFC_{comfort}$ has the best performance ever. This last setting, however, is very far from the best handling achieved by the $VFC_{handling}$ which has handling performance comparable to the original BMW suspension setup slightly improving comfort. The $VFC_{mix}$ presents as expected a compromise between the two extremal control logics, being as good as Skyhook concerning comfort, while outperforming it for handling. 

Figure 2 and Figure 3 show the results regarding the passage of the vehicle on the various shapes of a bump at different speeds, as shown below. The plane related to the maximum handling, $J_{hand}$, vs. the minimum peak acceleration monitored by the car body,$\;J_{peak}$, is shown in Figure 2. It is worth pointing out the technological limitation shown by all the control logics that are arranged parallel to the dashed line found with different passive designs. All four controls and in particular the $VFC_{comfort}$, $VFC_{mix}$ and Skyhook are able to minimize the maximum acceleration very effectively at the cost, however, of worsening the handling, which is comparable to that of the standard setup. 

Figure 3 shows the results of the handling versus the settling time of the vertical displacement after crossing the bump. This last diagram is very useful for the comfort analysis because it evaluates, at once, the instantaneous vertical acceleration and also the coupled pitch and heave behavior of the vehicle. As expected, $VFC_{handling}$ has the best performance, followed by $VFC_{mix}$. It is worthwhile stressing the separation between the performance provided by the Skyhook setup and $VFC_{mix}$, both exhibit a comparable Handling, while the settling time is much shorter for VFC. 

In conclusion, the $VFC_{mix}$ the strategy represents an excellent compromise of performance and comfort both in terms of an extra-urban road cycle and in mitigating the accelerations and settling times of the vehicle in the presence of road bumps.

## 2. The Control System and the Sensor Network

The proposed VFC is developed on an experimental platform which is based on Arduino with interfaced commercial sensors and is employed to control magneto-rheological suspensions. The choice to use Arduino is due to two main reasons, the first is the possibility of working on an open source and inexpensive platform to test different control strategies. The second reason is due to the flexibility of the Arduino system, which gives the possibility to expand the number of sensors and their type: the use of redundant sensors can indeed improve the estimation accuracy of the measurement of a vehicle’s dynamic motion, especially in the presence of many sources of disturbance that generally are involved with a car vehicle. The first architecture tested consists of four different types of sensors, i.e., accelerometers, gyroscopes, magnetometers, and potentiometers, linked by an ethernet network employed to connect them to a central control unit. Ethernet communication is important because it guarantees the modularity of the architecture: individual control units can be indeed connected or disconnected without disturbing the transmission speed of the data, thanks to a suitable hardware shield of the communication protocol, which is attainable by the Arduino architecture. An internet protocol (IP) address is assigned to each unit, which is able to communicate with other control platforms in a simple, yet robust, way.

**Figure 4:**
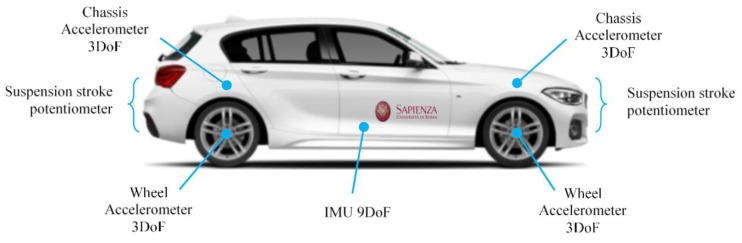
Sensors positions on BMW series 1.

Concerning this architecture, with reference to Figure 4, four linear potentiometers are employed, one is mounted on each suspension, together with a set of eight triaxial MEMS accelerometers, four of them installed on the wheels and the other four on the car body frame, near the joint of the suspension. The inertial measurement unit (IMU) with nine degrees of freedom, which consists of a three-axis accelerometer, a three-axis gyroscope and a three-axis magnetometer, is installed nearby the center of gravity of the car. The four sensors positioned on the frame have a twofold task, measuring the vertical accelerations near the suspensive attacks, and assisting the estimation of the overall vehicle attitude. In fact, the four 3-D accelerations acquired from the four corners are shared with the IMU to better estimate the pitch and roll of the vehicle. 

**Figure 5:**
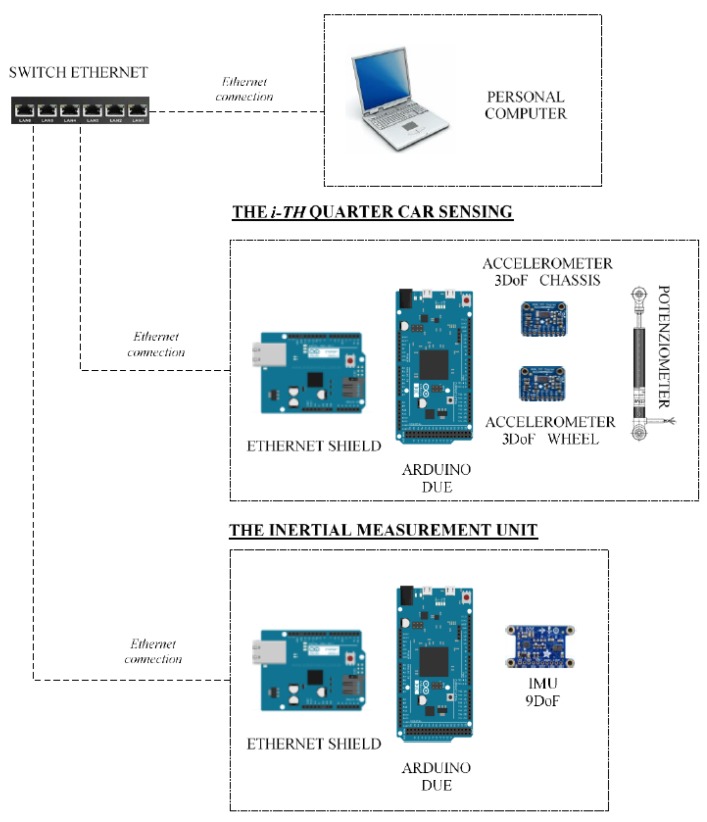
Sensors architecture and communication scheme.

**Figure 6:**
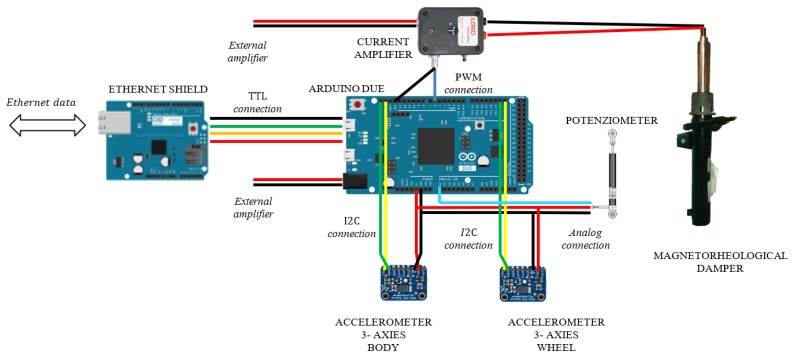
Complete diagram of electrical connection of the damping controller.

With reference to Figure 4 and Figure 5, the control hardware consists of five platforms of the type “Arduino Due”, which are linked to the mentioned sensors. Four Arduino boards are located at the four sides of the car and they are linked to two accelerometers, placed on the body and on the wheel of the car, and to a potentiometer, in order to record the stroke of the suspension. The fifth Arduino board is integral to the 9DOF (Degrees of Freedom) inertial platform, which makes it rigidly connected the car’s vehicle body. The four controllers arranged on the four sides of the vehicle, measure the data of the accelerometric and potentiometer sensors, have also the task of actively controlling the characteristics of the suspensions and are connected to current generators (see Figure 6 for more details). The set of Arduino boards, connected by ethernet modules and their switches, are connected to a dedicated PC, which is employed to impart control rules or strategies or simply for the real-time acquisition of the data. 

## 3. Optimal Control Strategy 

Recently the Mechatronics and Vehicle Dynamics research group of Sapienza university is working on the autonomous vehicle project and is developing different control strategies deriving from the principle of the optimum [22,23,24]. The VFC is a dynamic compensator that is obtained by the solution of Pontryagin’s minimum principle for nonlinear dynamical systems [25,26]. Through the calculation of the variations and with certain assumptions, the Pontryagin open-loop optimum problem can be roughly solved according to the local minimum principle with the possibility of generating a category of purely feedback controls. The novelty of the proposed mathematical model is based on the introduction of a special objective function, together with on certain, weak, assumptions on the form of the dynamic equation of the controlled system.

As a brief resume on optimal control theory, it can be summarized as, given non-linear dynamic system characterized by $\dot{x}=f\left(x,u,t\right)$, the optimal control J is an algorithm that optimizes, minimizing or maximizing, a certain objective function, which may depend on the state variables x such as on the input u. In symbols, it holds:

(1) $$\{\begin{array}{c} J=\int_{t_{0}}^{t_{f}}E\left(x\left(t\right),u\left(t\right),t\right)dt\;\;\;\;:\;\;\;\underset{u\in U}{Opt}J\;\;\\ \dot{x}\left(t\right)=f\left(x\left(t\right),u\left(t\right),t\right)\\ x\left(t_{0}\right)=x_{t_{0}} \end{array}\;$$

on u∈U, which sets the physical constraints of the control variables $u_{1}\left(t\right),\;u_{2}\left(t\right),\;\ldots ,\;u_{r}\left(t\right)$. In the maximum case, the Pontryagin solution $u^{*}$ specifies the condition:

(2) $$J\left(u\right)\leq \;J\left(u^{*}\right)\;,\;u^{*},u\in U$$

To fulfill the constraint condition $\dot{x}=f\left(x,u,t\right)$, the cost function is generally completed employing the Lagrangian multiplier $\lambda \left(t\right)=\;{\left[\lambda_{1}\left(t\right),\;\lambda_{2}\left(t\right),\;\ldots ,\;\lambda_{n}\left(t\right)\right]}^{T}$, which leads to:

(3) $$\{\begin{array}{c} \tilde{J}=\int_{t_{0}}^{t_{f}}E\left(x,u,t\right)+\lambda^{T}\left(\dot{x}-f\left(x,u,t\right)\right)\;\;dt\;:\;Opt\tilde{J}\;\;\\ x\left(t_{0}\right)=x_{t_{0}} \end{array}$$

The direct application of the Pontryagin technique produces the Euler-Lagrange equations in terms of x,u,λ, where $x_{t_{0}}$ is used for the initial conditions and $\lambda^{T}\left(t_{f}\right)$ for extremal conditions:

(4) $$\{\begin{array}{c} {\frac{\partial E}{\partial x}}^{T}-{\dot{\lambda }}^{T}-\lambda^{T}\frac{\partial f}{\partial x}=0\\ {\frac{\partial E}{\partial u}}^{T}-\lambda^{T}\frac{\partial f}{\partial u}=0\\ \dot{x}=f\left(x,u,t\right)\\ x\left(t_{0}\right)=x_{t_{0}}\\ \lambda^{T}\left(t_{f}\right)\delta x\left(t_{f}\right)=0 \end{array}$$

For a particular class of objective functions L(f,y), where y is the external sources and considering an affine non-linear dynamic system in the state x while is linear in the control u:

(5) $$\begin{array}{c} L\left(f,y\right)=f^{T}Af+f^{T}By\\ f\left(x,u,y\right)=\varphi \left(x,y\right)+S\left(x,y\right)u \end{array}$$

the optimal solution (4) leads the explicit feedback control:

(6) $$u={\left[\tilde{A}S\left(x,y\right)\right]}^{+}\left[-\tilde{A}\varphi \left(x,y\right)-By\right]$$

The matrices $A=\tilde{A}-A^{T}$ and B are the gains of the control to choose which state variable has to be minimized, φ(x,y) and S(x,y) are the non-linear component of the dynamic system f that has to be controlled, the exponent ${\left[\;\right]}^{+}$ means a pseudo-inverse matrix. 

Using this approach, a new class of variational controls is found, which can be proficiently applied for semi-active and active control systems, as it is discussed in the forthcoming section.

## 4. The Experimental Setup

### 4.1. A New Semi-Active Suspension Control

The VFC is applied to a quarter of the vehicle as described in [14] and is briefly recalled below for greater clarity.

**Figure 7:**
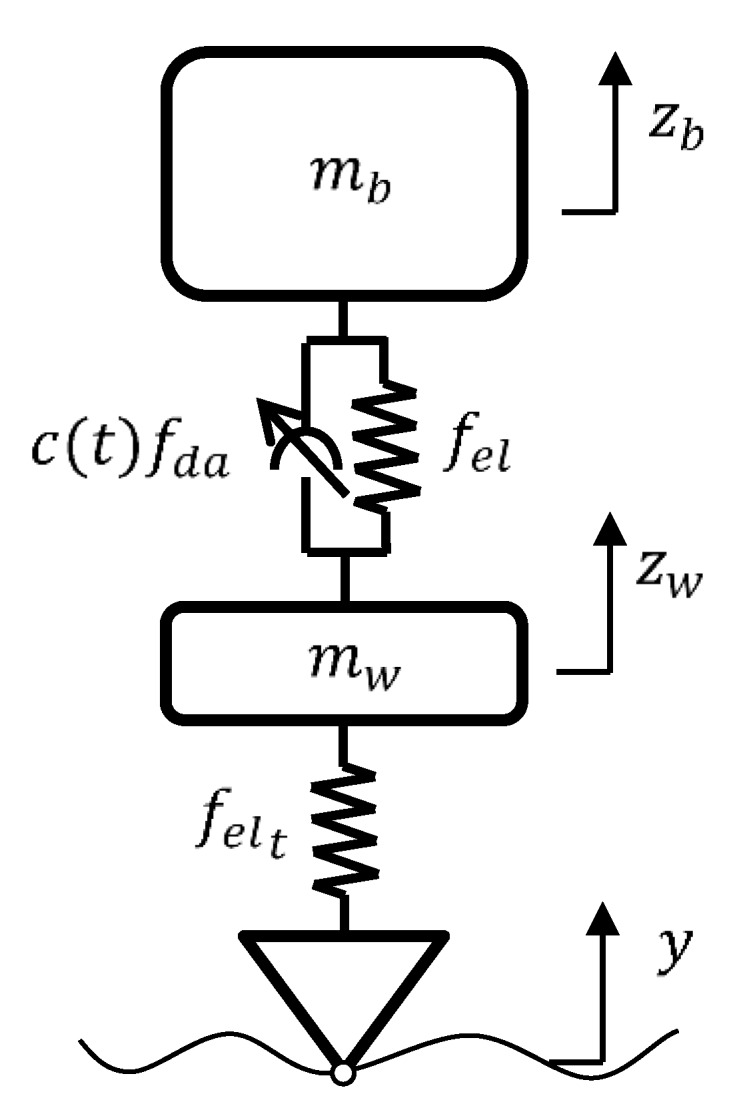
A full quarter suspension system with a controlled damper.

A full quarter suspension, shown in Figure 7, consists of two degrees of freedom system including the vertical motion $z_{b},\;z_{w}$ of the body and wheel with mass $m_{b}$ and $m_{w}$ respectively. The mechanical equations of motion, with the presence of the disturbance deriving from the ground y, are:

(7) $$\{\begin{array}{l} {\ddot{z}}_{b}+\frac{f_{el}\left(z_{b}-z_{w}\right)}{m_{b}}+c\left(t\right)\frac{f_{da}\left({\dot{z}}_{b}-{\dot{z}}_{w}\right)}{m_{b}}=0\\ {\ddot{z}}_{w}+\frac{f_{el}\left(z_{w}-z_{b}\right)}{m_{w}}+c\left(t\right)\frac{f_{da}\left({\dot{z}}_{w}-{\dot{z}}_{b}\right)}{m_{w}}+\frac{{f_{el}}_{t}\left(z_{w}-y\right)}{m_{w}}=0 \end{array}$$

where $f_{da}\left({\dot{z}}_{b}-{\dot{z}}_{w}\right)$, $f_{el}\left(z_{b}-z_{w}\right)$ and ${f_{el}}_{t}\left(z_{w}-y\right)$ could include geometric or constitutive non-linearity of the suspension system, i.e., damping and elastic devices, and the wheel elasticity ${f_{el}}_{t}$. The c(t) is the semi-active control variable. 

Proceeding as described in the general case of Section 3, we find the non-linear control law. In particular, reducing Equation (7) to the first order:

(8) $$\{\begin{array}{l} {\dot{x}}_{1}=-\frac{f_{el}\left(x_{2}-x_{4}\right)}{m_{b}}\;-\frac{c\left(t\right)}{m_{b}}\;f_{da}\left(x_{1}-x_{3}\right)\\ {\dot{x}}_{2}=x_{1}\\ {\dot{x}}_{3}=-\frac{f_{el}\left(x_{4}-x_{2}\right)}{m_{w}}\;-\frac{c\left(t\right)}{m_{w}}\;f_{da}\left(x_{3}-x_{1}\right)-\frac{{f_{el}}_{t}\left(x_{4}-y\right)}{m_{w}}\\ {\dot{x}}_{4}=x_{3} \end{array}$$

where

(9) $$\begin{array}{c} x=\left(\begin{array}{c} x_{1}\\ x_{2}\\ x_{3}\\ x_{4} \end{array}\right)=\left(\begin{array}{c} {\dot{z}}_{b}\\ z_{b}\\ {\dot{z}}_{w}\\ z_{w} \end{array}\right),\;\;\;\\ f=\left(\begin{array}{c} -\frac{f_{el}\left(x_{2}-x_{4}\right)}{m_{b}}\;-\frac{c\left(t\right)}{m_{b}}\;f_{da}\left(x_{1}-x_{3}\right)\\ x_{1}\\ -\frac{f_{el}\left(x_{4}-x_{2}\right)}{m_{w}}\;-\frac{c\left(t\right)}{m_{w}}\;f_{da}\left(x_{3}-x_{1}\right)-\frac{{f_{el}}_{t}\left(x_{4}-y\right)}{m_{w}}\\ x_{3} \end{array}\right) \end{array}$$

Using the decomposition of the system dynamics as for Equation (5), one obtains:

(10) $$\varphi =\left(\begin{array}{c} -\frac{f_{el}\left(x_{2}-x_{4}\right)}{m_{b}}\;\\ x_{1}\\ -\frac{f_{el}\left(x_{4}-x_{2}\right)}{m_{w}}\;-\frac{{f_{el}}_{t}\left(x_{4}-y\right)}{m_{w}}\\ x_{3} \end{array}\right),\;\;\;S=\left(\begin{array}{c} -\frac{f_{da}\left(x_{1}-x_{3}\right)}{m_{b}}\\ 0\\ -\frac{f_{da}\left(x_{3}-x_{1}\right)}{m_{w}}\\ 0 \end{array}\right),\;\;\;\;u=c$$

Applying the general solution (6) we obtain the non-linear control law as:

(11) $$\begin{array}{c} c\left(t\right)=sat_{c\left(t\right)\in \left[c_{min};c_{max}\right]}\{g_{0}\frac{f_{el}\left(z_{b}-z_{w}\right)}{f_{da}\left({\dot{z}}_{b}-{\dot{z}}_{w}\right)}+g_{1}\frac{{f_{el}}_{t}\left(z_{w}-y\right)}{\;\;f_{da}\left({\dot{z}}_{b}-{\dot{z}}_{w}\right)}+g_{2}\frac{{\dot{z}}_{b}}{\;\;f_{da}\left({\dot{z}}_{b}-{\dot{z}}_{w}\right)}\\ +g_{3}\frac{{\dot{z}}_{w}}{\;\;f_{da}\left({\dot{z}}_{b}-{\dot{z}}_{w}\right)}+g_{4}\frac{y}{\;f_{da}\left({\dot{z}}_{b}-{\dot{z}}_{w}\right)}\} \end{array}$$

where the *g*’s gains are tuning parameters related to the matrices A and B coefficients. The control law of the damping is expressed in the feedback form, as a function of the state vector. The $sat_{c\left(t\right)\in \left[c_{min};c_{max}\right]}\left\{\;\right\}$ is the saturation function that is applied when c falls outside the range $\left[c_{min};c_{max}\right]$, which is actually attainable by the damper.

Equation (11) defines the optimal version of the control, which is ready to be used once input data from suitable sensors are gathered. However, it should be noticed that the knowledge of the entire state of the system is attainable only in a few cases when very sophisticated and costly measurement instruments are adopted. Therefore, the approach followed here, which is more general, is to rely only on those inputs that can be effectively measured by the available onboard sensors, such as that described in the previous section, and, when required and/or possible, the state variables that are not easily amenable for measurements are estimated by data fusion techniques. Due to the cost and commercial availability limitations, our BMW series 1 was equipped with accelerometers, potentiometers, and IMU. Therefore, while the suspension deflection $\left(z_{b}-z_{w}\right)$ is directly measured by the potentiometer, its first-time derivative in equation (11) has to be estimated. In the same fashion, the moduli of the variables ${\dot{z}}_{b}$,$\;{\dot{z}}_{w}$,$\;z_{b}$ and $z_{w}$ are predicted by integrating and filtering, e.g. Kalman filter, the data acquired from the accelerometer and the gyroscope; these concepts will be discussed in detail in the forthcoming sections. The road asperities y are usually measured through surface detection sensors, such as cameras or laser systems, which are devices out of the scope of this first testing phase: therefore, the variable *y* remains unknown. 

In summary, the sensors mounted onboard the BMW 1 series in Figure 4 can acquire the following variables:

(12) $$a\prime_{b_{i}}\;,\;a\prime_{t_{i}},\;{\Delta \prime }_{i},\;\omega \prime ,\;h\prime$$

where $a^{\prime }=\left[\ddot{x}\prime \;,\;\ddot{y}\prime \;,\;\ddot{z}\prime \right]$ is the vector of the accelerations measured on the body frame along the three axes; the subscripts *b* and *t* stand for body and tire; primes denote the vector components in the body reference frame; the index i∈[1,2,3,4] is used to locate the four positions of the sensors, respectively: 1 for front right, 2 for front left, 3 for rear right and 4 for rear left; Δ′ is the suspension stroke; $\omega^{\prime }$ and  h′ are the angular velocity vector acquired by the gyroscope and the direction and intensity of the magnetic field, both computed in the body reference frame.

**Figure 8:**
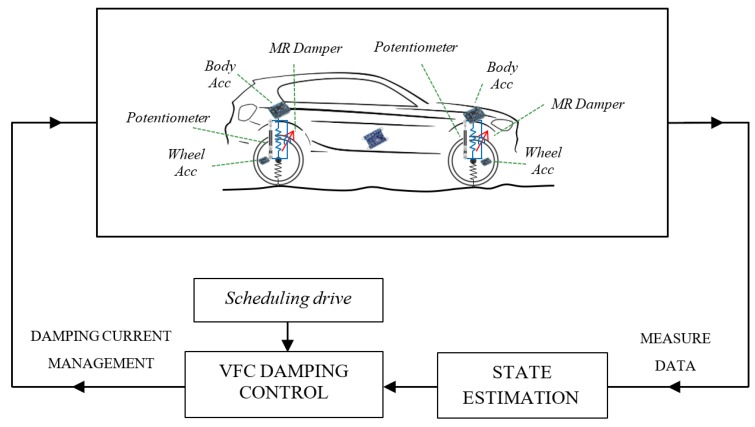
Sensors and control diagram for the magneto-rheological damper.

**Figure 9:**
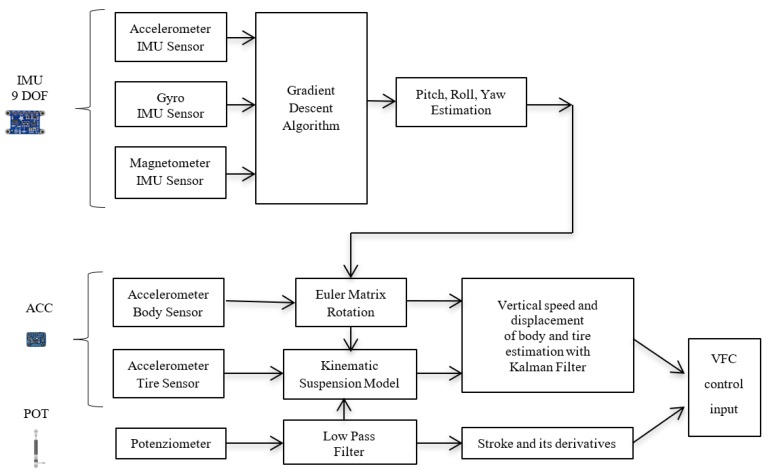
Data fusion and filtering diagram for variational feedback control (VFC) data input.

Figure 8 shows the control diagram for magneto-rheological damper: the measure data (12) gathered by the embedded sensors are used as input in the VFC controller (11) that manages the current generated by amplifiers. The state estimate includes a series of filters and data fusion briefly reported in the diagram in Figure 9. Through an accurate selection of the gains, it is possible to select the sport, comfort or mix driving style by activating different VFC settings stored in the scheduling drive box.

### 4.2. The Instrumented Car 

**Figure 10:**
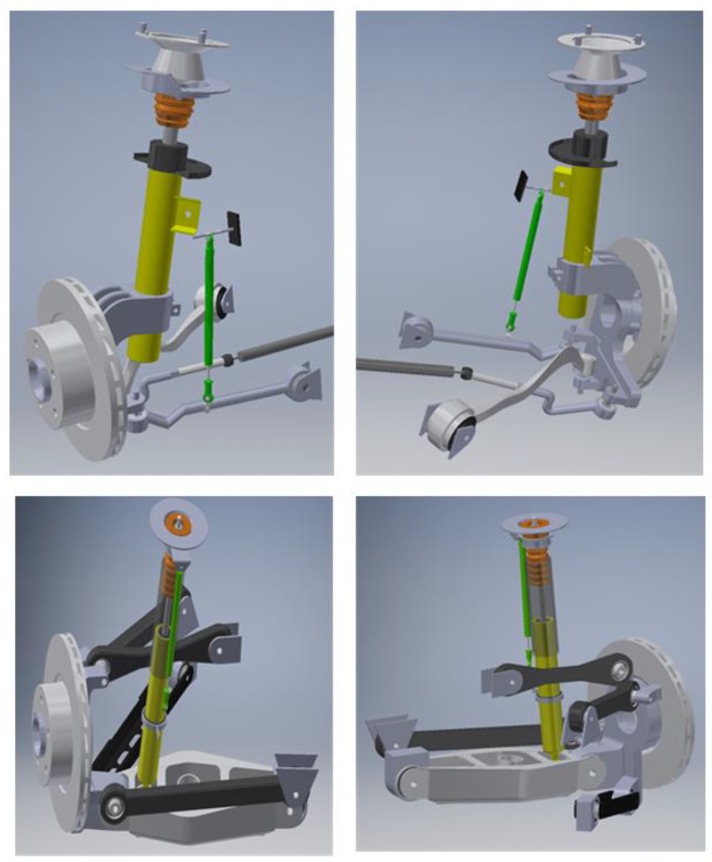
CAD of the original BMW front kinematic suspension at the front (top) and the rear (bottom).

**Figure 11:**
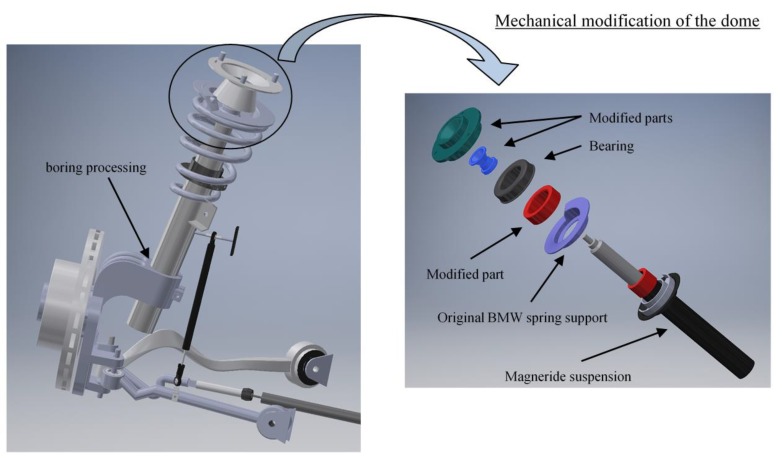
CAD of the front *Magneride* suspension and modifications to the upper dome.

**Figure 12:**
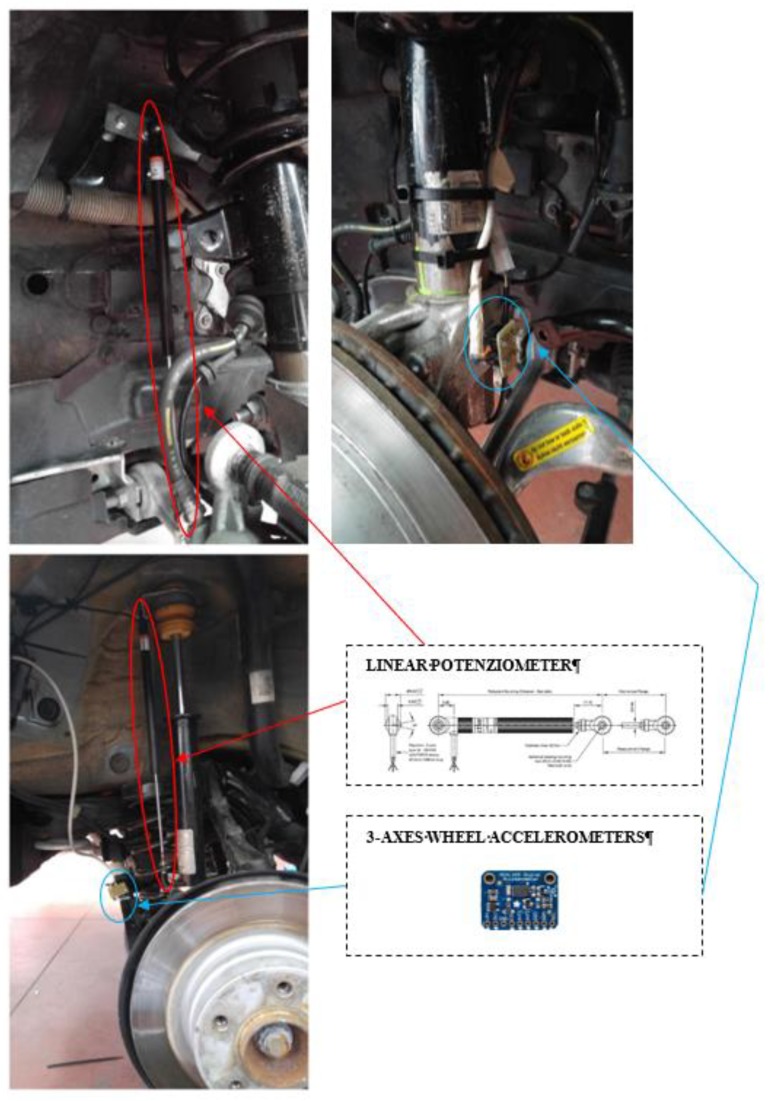
Linear potentiometer sensor and accelerometer are installed on front and rear suspension.

The sensor setup and the VFC controller described in the previous section and outlined in Figure 4 are mounted on board the real car, a BMW Series 1 E87, a test car that belongs to Sapienza Laboratory. The standard car was originally equipped with passive suspensions, which were replaced with magneto-rheologic suspensions after the suspension setup was completely re-engineered for the modifications required (see Figure 10, Figure 11 and Figure 12 for details). The *Magneride™* [27] suspension is the magneto-rheological (MR) damper system chosen for the project. 

The main physical and geometrical properties of the BMW series 1 were experimentally measured and the values are listed in Table 1; these values will be the parameters employed within the dynamic equation and within the control law introduced in the previous section, to run numerical experiments for the tuning of the VFC control logic. The position of the center of gravity of the car is measured by load cells, the elastic stiffness of the suspension is measured with linear potentiometers, both are installed on the suspensions as shown in Figure 12. The rigidity of the front and rear spring were constant as the stroke varied. The estimation of the elastic characteristics was performed both by the analysis of the finite elements of Ansys and by experimental tests conducted with compression and expansion values of the suspension far from the end-of-travel limits. The procedure for the damping estimation requires dynamic experiments that are explained in Section 5.

The front suspension was originally equipped with a multi-link MacPherson strut, which is a sophisticated structure where the usual triangular bottom link is replaced with two transverse links with spherical joints. These links connect the wheel-carrier to the frame. The rear suspension is a five links system with spherical joints (Figure 10).

The kinematic solution of the suspension setup is needed since it is fundamental to identify the transfer function between the difference of the vertical displacement of wheel and the car body to correlate the potentiometer output with the stroke of the suspension. In this regard, reverse engineering was applied to the suspensions, so that CAD models, depicted in Figure 10, and the kinematic solution is provided.

### 4.3. The Magneride Suspension

**Figure 13:**
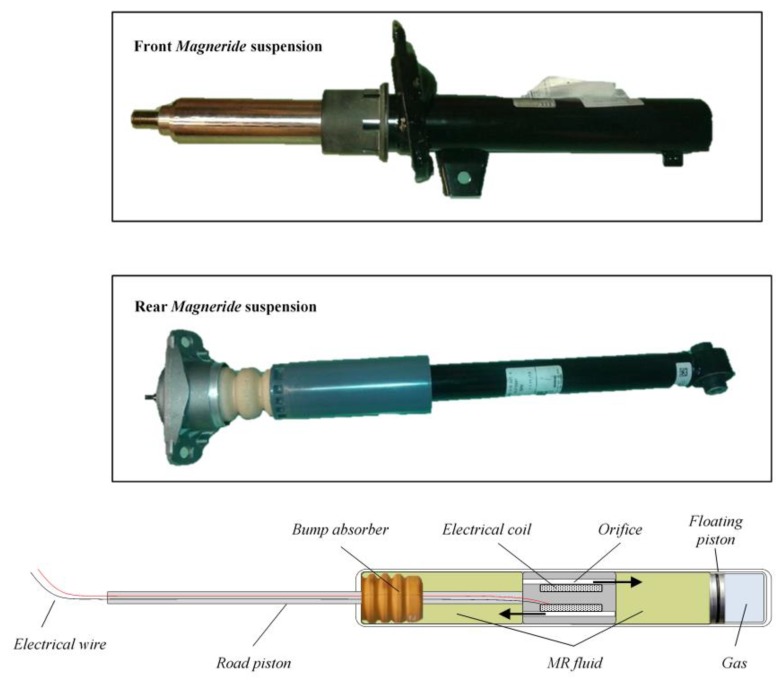
The magneto-rheological damper installed on BMW (*MagneRide* of BWI Group).

The *Magneride*, produced by BWI [27], is selected to develop the VFC and, after in-depth kinematics, dynamics and structural analyses, were mounted into the BMW, changing its suspension system. As shown in Figure 13, *MagneRide* has no mechanical valves or small moving parts, since the shock absorber is a single-tube damper with a floating piston De Carbon-like architecture, it employs a MR damper fluid filled with minute metallic particles, which can be rapidly and provisionally magnetized by the application of a current through the electrical coils in the damper piston. In the standard state, in absence of current application, the metallic particles are randomly scattered within the fluid, so that the damper is in its softest setting. When a certain amount of current is applied, the metallic particles are magnetised and attract each other, with a force of attraction that depends on the intensity of the magnetic field: this increases shear stresses within the flow and, as a final result, increases the damping. In this way it is possible to change, almost immediately and continuously, the damping force simply changing the applied current; the power absorbed by each damper is low, less than 20 W. More information on how the magneto-rheological suspensions work can be found in the following references [28,29,30,31,32]. The first application of *MagneRide* was reported in the Cadillac Seville STS (2002) produced by General Motors, this setup is now employed, as standard or optional equipment, in several US models from GM vehicles, such as Buick, Cadillac, Chevrolet, and others. It was also adopted on some non-US vehicles such as Ferrari, Audi and Holden Special Vehicles.

Concerning our experimental setup, an amplifier managed by the Arduino board is used to control *MagneRide*. The selected amplifier is produced by LORD (Cary, NC, USA) [33] and it can change the current flowing through the shock absorbers in such a way that the damping is varied in real time. With reference to Figure 6, the amplifier consists of: (i) a power supply input at 12 V DC; (ii) output connectors through which the current is sent to the damper; (iii) a Bayonet Neill–Concelman (BNC) terminal to input a Pulse-Width Modulation (PWM) control signal at about 1 kHz, managed by the Arduino board.

With reference to Figure 11, in order to adopt *MagneRide* in the damper of the BMW, the inner diameter of the wheel-carrier had to be changed, changing also the lodging of the spring, to work with the same static deflection and therefore to operate in the same condition as with the original setup. In addition, also the upper dome had to be entirely redesigned. Regarding the rear suspension, the lower end was modified to install *MagneRide*, by replacing the pre-existing hinge with a spherical joint for the multi-link kinematics.

### 4.4. Hardware and Sensors Specifications 

With reference to the Figure 5, Figure 6 and Figure 12, the main characteristics of the sensors mounted on board of the BMW 1 Series are discussed below:

1-Potenziometer sensor: the MLS-0952 (Motorsport Linear Sensor) is a linear, limited noise potentiometer, with a maximum attainable speed of about 10 m/s. It is connected to the 5 V DC power supply to permit to be measured by the electric logic level of Arduino.

Car body and tire accelerometer sensors: the ADXL345 is a 3-axis MEMS accelerometer, characterised by low power, high resolution (13-bit) measurement at up to ±16 g. The digital output data is the I2C digital interface. Its high resolution (4 mg/LSB, where LSB is the Least Significant Bit) enables measurement of inclination changes less than 1.0 degrees.

Inertial measurement unit sensor: The inertial-measurement-unit chosen is Adafruit 9-DOF, made of three sensors: a 3 axes of accelerometer, a 3 axes gyroscopic and a 3 axes magnetic (compass). The L3DG20H is the gyroscope hardware and LSM303DLHC is the accelerometer and compass hardware. All of them use I2C and is possible to communicate with all of them using only two wires with a resolution up to 13-bit. The L3GD20H 3-axis gyroscope is a type of sensor that can sense twisting and turning motions up to ±2000 degree-per-second scale. Inside the LSM303DLHC there are two sensors, one is a classic 3-axis accelerometer, the other is a magnetometer that can sense where the strongest magnetic force is coming from used to detect magnetic north. LSM303DLHC has linear acceleration full-scales up to ±16g and a magnetic field full-scale up to ±8.1 gauss.

Controllers: with reference to Figure 5 and Figure 6, the connection diagrams of the Arduino controllers with the sensors are reported. In particular, the reference platform used is Arduino Due, a board that operates in 32-bit to achieve the maximum performance. Overall, five Arduino boards are installed, four of which are placed next to each wheel to measure data form the accelerometers mounted on the body and on the wheel and to measure data form the potentiometer, and also to control the current amplifier that modifies the damping characteristics of the MR. The central Arduino board is connected to the 9 DOF IMU, and its purpose is to compute the attitude of the vehicle through filtering and data fusion algorithms. Figure 6 documents the analog connection with the potentiometer with a resolution of a tenth of a millimeter; an I2C link with the two ADXL345 accelerometers, which permits long wiring and the stability of communication through purposely sized pull-up resistors; a PWM connection for the control of the current sent to the MR damper. Figure 6 shows also the link with the 9 DOF IMU platform, which is I2C as well. As previously mentioned, all the boards have a specific ethernet shield to communicate with one another and a dedicated PC through the ethernet User Datagram Protocol (UDP).

### 4.5. Data Fusion and Filtering

Data gathered from different types of sensors is purposely analysed by suitable algorithms, in order to gain information concerning the variables of the system state that cannot be observed. 

The data fusion method used here combines the data acquired with the IMU inertial platform, to evaluate the position and kinematic parameter of the vehicle, starting from the measure of the accelerometer. With reference to Figure 9, a complete diagram of the data fusion and filtering operation is shown to prepare the VFC input data. The gradient descent algorithm for IMU is here used, to provide a direct estimation of pitch roll and yaw employing the nine degrees of freedom of the IMU platform [34]. The estimated pitch, roll and yaw are employed to rotate the data measured by the accelerometer in the body reference frame, in order to convert it into to the fixed reference frame: the vertical acceleration is extracted and the gravitational acceleration measured by the MEMS sensors is filtered out, since it would produce a drift of the vertical speed and displacement, when the acceleration is integrated. To conclude, from the knowledge of the orientation of the car body, using the data acquired by the potentiometer, together with the kinematic laws of the suspension, it is possible to evaluate also the absolute vertical speed and displacement of the tire, necessary to implement the VFC control

## 5. Fine Regulations of the Variational Feedback Control (VFC)

The four Arduino boards that govern the four suspensions are used to implement and perform the fine tuning of the VFC control law. With reference to Equation (11), gains within the control equation pay the role of tuning parameters and are not selected a priori, since VFC is employed to control nonlinear systems, therefore a closed form relation between gains and the system response is not generally attainable. In fact, the particular objective function used in the variation method (5) requires minimizing the entire dynamic function in a quadratic form. This request means that the control has a strong coupling between the coefficients of the gain matrices and the dynamics itself. Therefore, a direct correlation between handling/comfort and the gains of the control is not assured and a tuning procedure to regulate the feedback control is needed.

Concerning the tuning of the gains, the vehicle is numerically modeled in order to implement and test the proposed control logic the gains are identified employing a genetic algorithm (GA), that is, a heuristic method of optimizing problems [35]. Solutions provided by GA to the optimization problems are based on techniques inspired by natural evolution, such as inheritance, mutation, selection, and crossover: the technique employed to find the best values of the gains minimizes an objective function, which are the ideal indicators as introduced at the beginning of the paper. As an example, once the best comfort is required, the control evaluation function that provides the optimal gains is defined ahead:

(13) $$J_{comf}=\sigma_{{\ddot{z}}_{b}}$$

**Figure 14:**
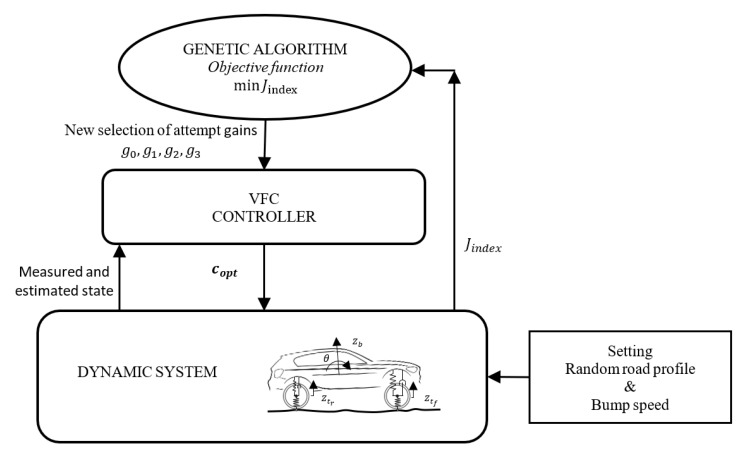
Genetic algorithm applies to the gains in VFC identification.

Thanks to the use of GA, they randomly produce a starting guess for the gains and then runs the simulation of the VFC-controlled model, so that the time history of the acceleration is computed, its variance is evaluated and, finally, $J_{comf}$ is known. At this point, GA generates a new set of gains, according to how much the $J_{comf}$ improves or worsens, with specific techniques of the algorithm, as shown in Figure 14. Convergence or the reaching of the absolute minimum are not guaranteed for GAs, however, they are among the best methods that can proficiently deal with non-linear systems. The control values selected by the GA algorithm are of three types as seen in Section 1: i) gains are selected to optimize handling in general, ii) gains are selected to improve comfort and iii) an intermediate gain selection which is a compromise between the previous two. The selection of gains has been performed on two types of tracks, a rough road of an extra-urban profile, as per regulation [36], and a bump speed profile.

Results of the application of GA algorithm are obtained from the simulation of a dynamic half-car model, where the stroke-end of the suspension was also considered since it is a fundamental feature of the suspension behavior that is very important to consider for optimal results in real life applications. The dynamic equations of motion for the half-car model are stated ahead:

(14) $$\begin{array}{l} m_{b}{\ddot{z}}_{b}=-k_{f}\left(z_{b}+l_{f}\theta -z_{wf}\right)-k_{r}\left(z_{b}-l_{r}\theta -z_{wr}\right)-c_{f}\left(t\right)\left({\dot{z}}_{b}+l_{f}\dot{\theta }-{\dot{z}}_{wf}\right)-c_{r}\left(t\right)\left({\dot{z}}_{b}-l_{r}\dot{\theta }-{\dot{z}}_{wr}\right)\\ J\ddot{\theta }=-l_{f}k_{f}\left(z_{b}+l_{f}\theta -z_{wf}\right)+l_{r}k_{r}\left(z_{b}-l_{r}\theta -z_{wr}\right)-l_{f}c_{f}\left(t\right)\left({\dot{z}}_{b}+l_{f}\dot{\theta }-{\dot{z}}_{wf}\right)+l_{r}c_{r}\left(t\right)\left({\dot{z}}_{b}-l_{r}\dot{\theta }-{\dot{z}}_{wr}\right)\\ m_{wf}{\ddot{z}}_{wf}=-k_{tf}\left(z_{wf}-y_{f}\right)-k_{f}\left(z_{wf}-z_{b}-l_{f}\theta \right)-c_{f}\left(t\right)\left({\dot{z}}_{wf}-{\dot{z}}_{b}-l_{f}\dot{\theta }\right)\\ m_{wr}{\ddot{z}}_{wr}=-k_{tr}\left(z_{wr}-y_{r}\right)-k_{r}\left(z_{wr}-z_{b}-l_{r}\theta \right)-c_{r}\left(t\right)\left({\dot{z}}_{wr}-{\dot{z}}_{b}-l_{r}\dot{\theta }\right)\\ \Psi \left(z_{b},\theta ,z_{wf},z_{wr}\right)=0 \end{array}$$

where the subscripts, wf, wr, tf, tr,b, t, f, r are for the front and, rear wheel, front and rear tire, body, tire, front and rear, respectively, the coordinate $z,\;\dot{z},\;\ddot{z}\;$ are for the displacement and its first and second derivatives, $m_{b}$ is the weight of the car, $m_{wf},m_{wr}$ are the wheels masses, J is the momentum of inertia; l is the distance between the centrum gravity and the suspension link; θ is the pitch coordinate and y is the road surface; k is the stiffness elastic element and finally c(t) is the variable damping. The last equation Ψ is an algebraic constraint representing the stiffness increase due to the suspension stroke end. 

**Figure 15:**
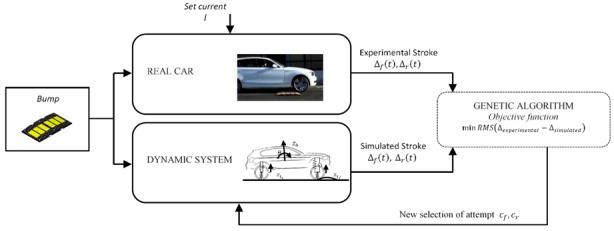
Genetic algorithm (GA) applied to the identification of the map I(c)*,* current vs. damping.

**Figure 16:**
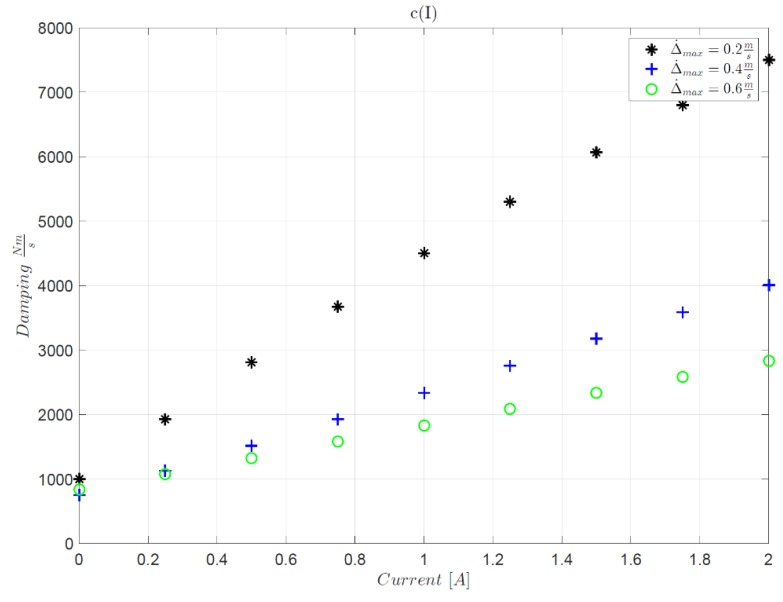
The map I(c), current vs damping for different crossing speed.

The optimization of the VFC is then followed by the process of identifying the correlation map that exists between the desired damping factor and the actual current injected into the *Magneride* suspension. It is interesting to mention that there are studies, such as in [11], that through the use of sophisticated laboratory equipment, are able to estimate the damping force of the damper through combined feed forward and feedback techniques to estimate the MR damper current for a given desired control force. With this information a mixed feedback-forward control system can be developed. However, in our case, parametric maps of the BWI dampers are not available, and the damping coefficient was estimated in an approximate way using a feed forward method by directly exploiting the experimental vehicle. Figure 15 shows the block graph related to I(c) map identification technique, where a genetic algorithm identifies the forward and rear damping value by minimizing the difference between simulated and experimental suspensive strokes Δ. Experimental activities were carried out concerning the crossing of a speed bump at different constant speeds keeping the current of *Magneride* constant. Figure 16 shows how the GA identifies the best damping factor associated with the injected constant current. The experiments led to a linear interpolation between the current and the damping and their ratio increases as the bump crossing speed increases. The higher the maximum compression speed ${\dot{\Delta }}_{\max}$ of the suspension and the greater the damping capacity of the damper for the same current. The map provides the relation between current and damping in function of the compression and rebound speed of the suspension, which in this case is linearly interpolated from the tests carried out in Figure 16.

## 6. Experimental Results

**Figure 17:**
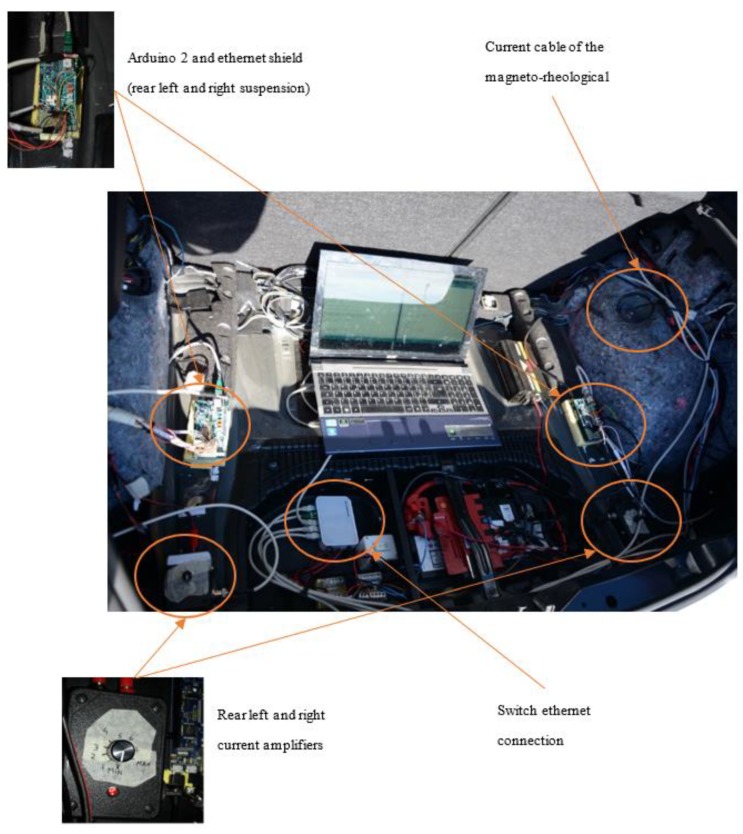
Organization of suspension control devices in the rear luggage compartment.

The experimental activity is aimed at showing how different gains for the VFC control logic can produce different results both in terms of power spectral density and time domain transient signals of the response. Correspondently, two different suspension responses are analysed in the experimental campaign: (i) statistical evaluation of comfort and handling on extra-urban tracks; (ii) transient response to bump crossing. The results shown are acquired directly acquired from the on-board instrumentation via a central computer. Figure 17 shows the layout of the control boards for the rear suspension with the wiring, the Ethernet network, and the controlled current amplifiers details. 

**Figure 18:**
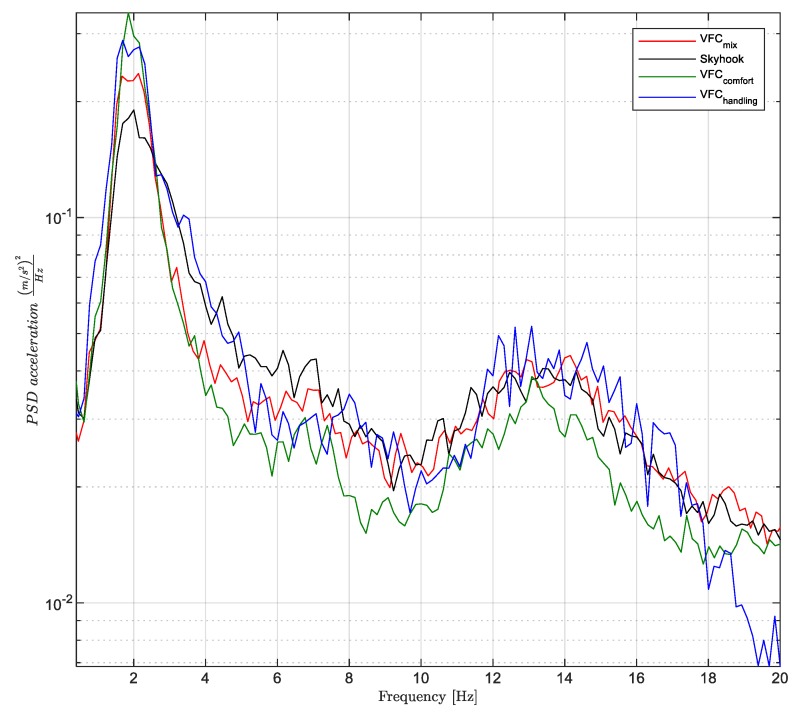
Power spectral density (PSD) of the vertical chassis acceleration for comfort evaluation.

With reference to the extra-urban road test, the results are summarized in Figure 1. They summarize several maneuvers, with different road-input, such as bumps, bents, braking, accelerations, random paths on fast roads. Figure 18 shows the power spectra densities (PSDs) of the vertical acceleration of the car body $S_{{\ddot{z}}_{b}}\left(f\right)$ obtained for three different control settings of the VFC and for the skyhook control. The purpose of the suspension in this context is to minimize the discomfort of the passengers, which obviously involves a minimization of some measure of the vehicle body motion, by the damping control. The assessment of the best comfort is a subjective opinion as it depends very much on the response of each individual. There are numerous studies in this regard that analyze the response of human subjected to excitations with constant frequencies and are studied how the amplitude and duration of oscillation determines a state of discomfort or not [37]. As far as the vertical accelerations linked to land transport are concerned, the study of comfort is analyzed in three frequency bandwidths: (i) the low frequency band <1 Hz in which mainly the human suffers from nausea, stomach pain and vomiting; (ii) the band between 1–2 Hz which is generally not associated with motion sickness problems although they may cause impaired skilled performance, inefficiency and fatigue; (iii) and the bandwidth between 2–20 Hz which is associated to fatigue discomfort and bad feeling of the passengers about the car insulation from the road. The latter bandwidth is associated to many natural frequencies of the human internal organs with the exception of the stomach. 

Now, analyzing Figure 18, we can focus on the evaluation of the different controller’s behaviors. The *VFC-comfort* setting reduces the vertical accelerations of the vehicle more effectively than the skyhook. The *VFC-mix* produces a lower response in the 2–20 Hz frequency bandwidth. Instead, around the first natural frequency of the vehicle, i.e., 2 Hz, the skyhook control shows its lowest value. As far as *VFC handling* is concerned, the comfort indicator worsens and decays in the 12–18 Hz bandwidth set by the natural frequency of the wheel, while the *VFC mix* has an intermediate trend between the other two.

**Figure 19:**
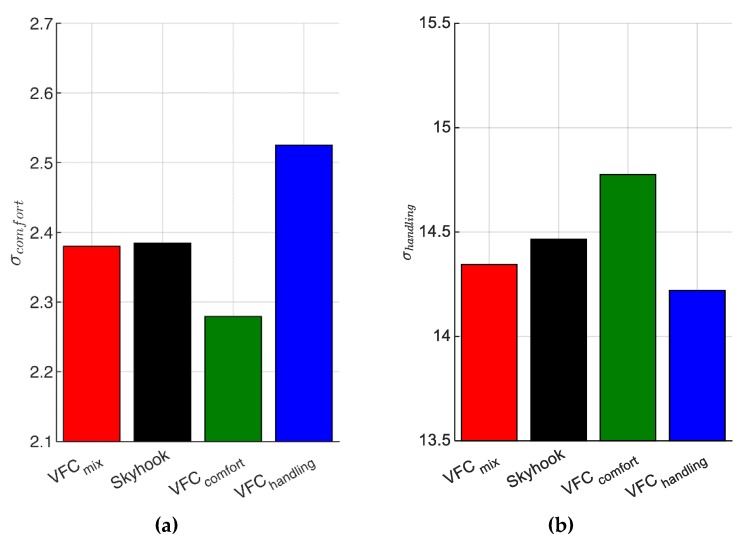
(**a**) The standard deviations of the vertical acceleration [$m/s^{2}$] and (**b**) suspension stroke [mm].

In order to globally evaluate comfort, it is usual to compare the standard deviations of vertical accelerations in the 0–20 Hz frequency band of the different controllers [18]. The quality index $J_{comfort}$ evaluated in Figure 1 is associated with the standard deviation $\sigma_{comfort}$ so calculated:

(15) $$\sigma_{comfort}=\sqrt{\int_{0\;Hz}^{20\;Hz}S_{{\ddot{z}}_{b}}\left(f\right)df}$$

where $S_{{\ddot{z}}_{b}}\left(f\right)$ is the PSD of the vehicle vertical acceleration. Figure 19a shows, in brief, the results of $\sigma_{comfort}$ obtained for the four controls.

**Figure 20:**
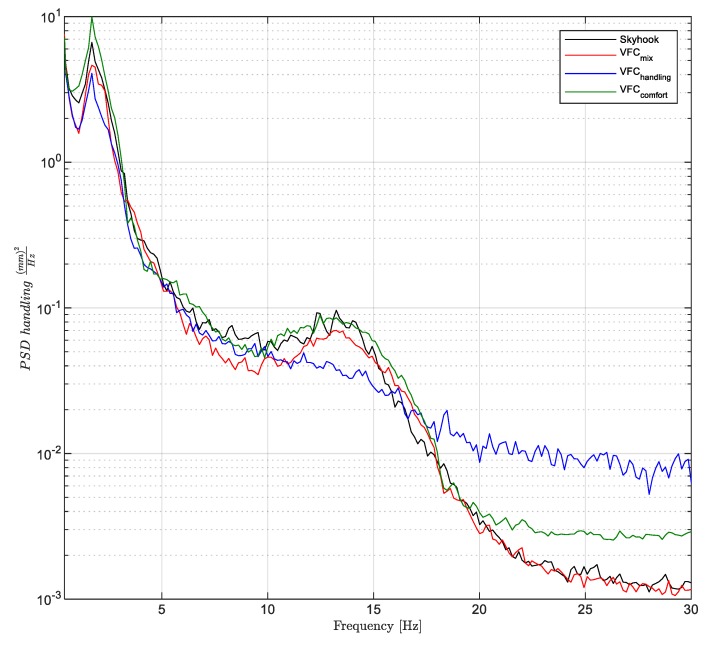
Power spectral density of the rear suspension stroke of the extra-urban track.

Vice versa, the integral of the PSD’s curves shown in Figure 20, are associated to the statistics of the handling level response. The road holding, i.e., the quality index $J_{handling}$, is calculated through the integral of the PSD of the suspension stroke $S_{\Delta }\left(f\right)$ between 0 and 30 Hz, and the result is summarized in the bar graph shown in Figure 19b.

(16) $$\sigma_{handling}=\sqrt{\int_{0\;Hz}^{30\;Hz}S_{\Delta }\left(f\right)df}$$

Figure 20 shows how the *VFC handling* curve is the lowest in absolute terms, which confirms that the *VFC-handling* is the lowest $\sigma_{handling}$ standard deviation. The latter mitigates both the vertical response at the vehicle first natural frequency and at that of the wheel. However, an increase of the *VFC-handling,* in the higher frequency response (after 18 Hz) is observed. Vice versa, the *VFC-comfort* has the worst trend in the range of the vehicle’s first natural frequency. The remaining two control logics have almost similar trends, and both show the highest oscillation in the 2 Hz frequency range.

The two bar graphs of Figure 19 summarize the results of Figure 1 and the Table 2 shows the gains used by the control logics used in the experimental setup.

**Figure 21:**
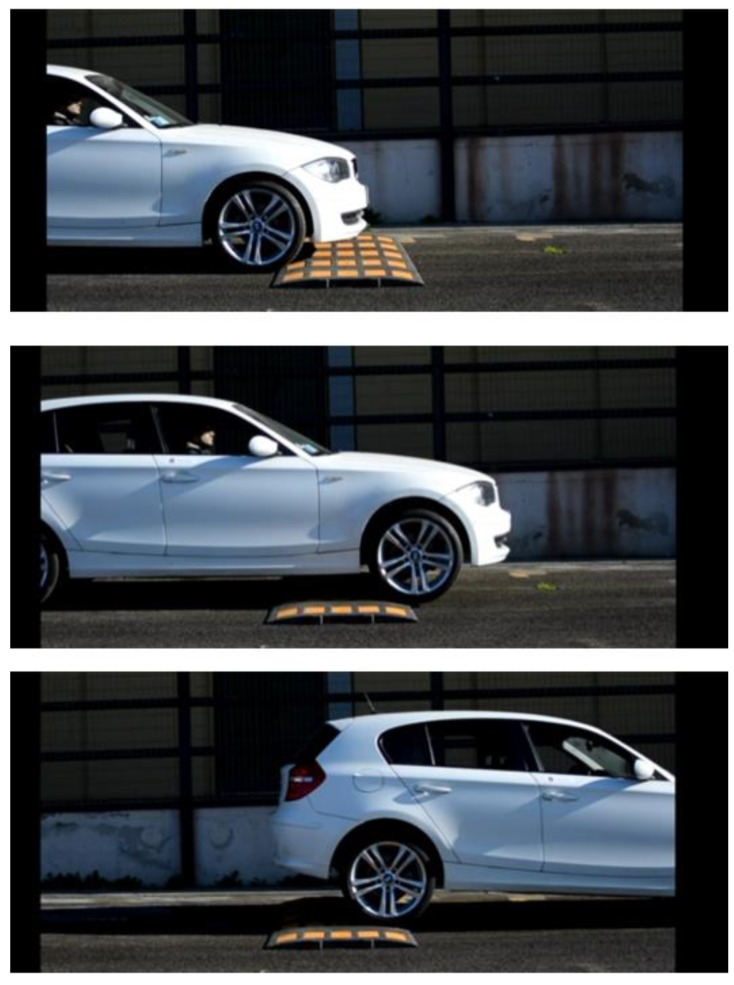
Experimental test of bump speed crossing.

**Figure 22:**
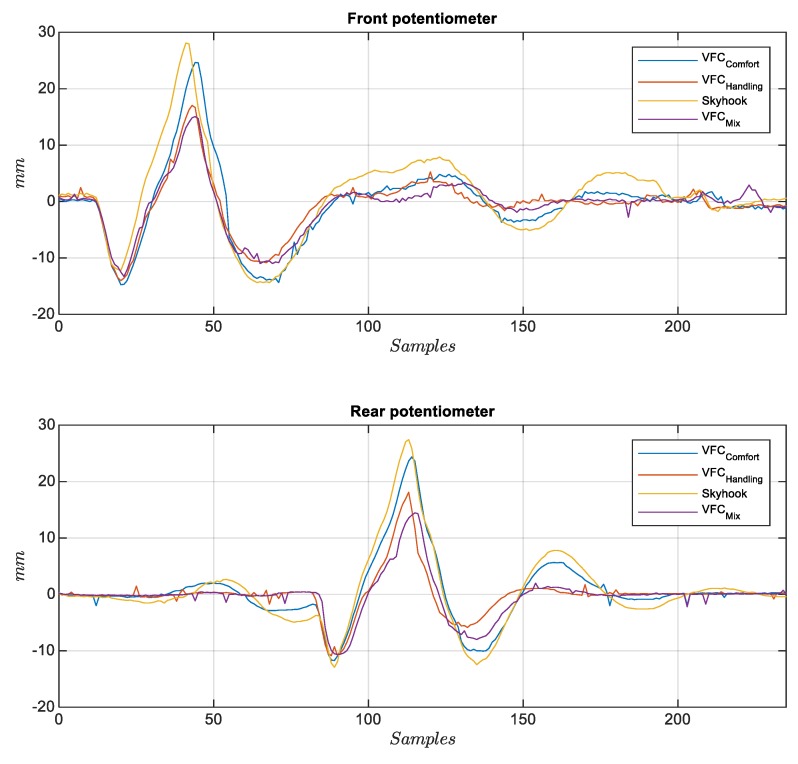
Rear (bottom) and front (top) potentiometer crossing a speed-bump for different controls.

**Figure 23:**
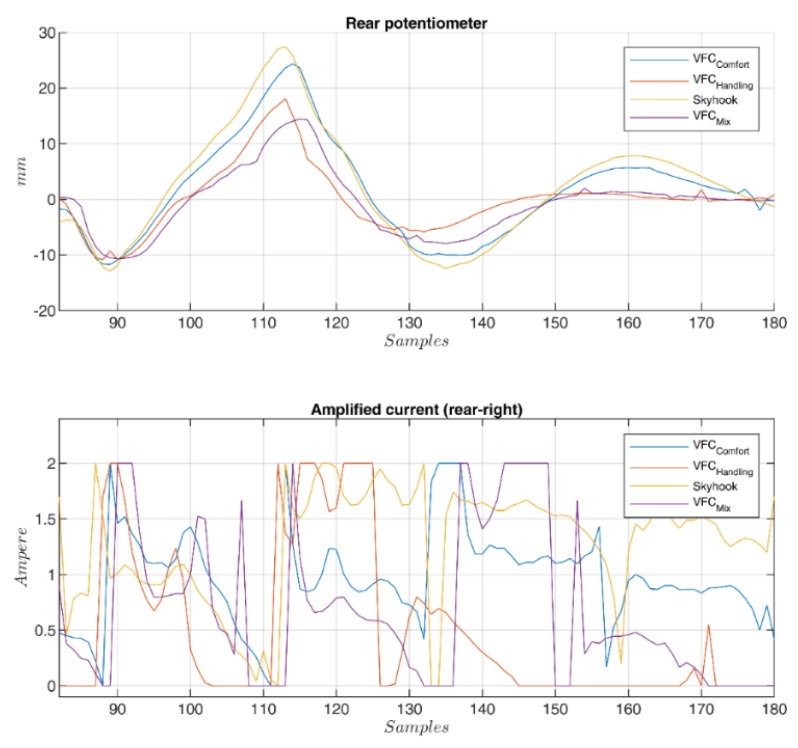
Magnification of the rear stroke (top) and its amplified current (bottom) crossing a speed-bump for different controls.

The second analysis concerns the passage of the vehicle on speed bump at different speeds (see Figure 21) to verify the behavior of the suspensions subjected to strong shocks. Many tests were performed with different bump profiles and at different speeds, results are summarized in Figure 2 and Figure 3 and in Figure 22 where the acquired trend of the potentiometers during a passage is reproduced. The subplot on top in Figure 22 shows the front potentiometer, while the rear one is shown in the subplot on the bottom. Among all four controls, both *VFC handling* control and the *VFC mix* exhibit good damping and limited settling times, the excursion is indeed the lowest with the *VFC mix* followed by *VFC handling*, while the *VFC comfort* and the *skyhook* have the largest excursions. Moreover, the amplified input current in the rear damper is shown in Figure 23, with a comparison of the different control strategies.

## 7. Conclusions

The paper is focused on recent experimental activity on the semi-active suspension control. The new plant is characterized by a network of sensors that, cooperating with a new control approach developed in the context of optimal control theory by the authors, drives a system of magneto-rheological actuators. The control method is based on a variational technique of Pontryagin’s type. The basic theory of the method, called variational feedback control (VFC) is employed and fed by the data acquired by a large set of sensors deployed at key locations on board the car. A suitably modified BMW series1 car equipped with semi-active dampers is used as a technology demonstrator. An Arduino-based hardware architecture is interfaced with the network of sensors and with the magneto-rheological actuators, allowing comfort and road holding to be controlled with low-cost and easy-to-install electronics. Four separate controllers drive the four suspensions by sharing the data with each other to improve the estimate of the state of the vehicle through the ethernet network of sensors. After a careful experimental campaign aimed at tuning the various control strategies, the data, in different driving conditions outside the city, have been collected. The results show large flexibility of the system that is capable of merging opposite suspension requirements, related both to comfort and handling. The different VFC settings allow you to switch from a sporty driving, in which road holding abilities are enhanced, to maximum driving comfort.

## Figures and Tables

**Table 1:** Main characteristics of the vehicle and passive suspensions.

Main Physical and Geometrical Properties of the Vehicle	Values
Vehicle weight (Measured with passengers)	1,530 kg
Front tire mass (estimated)	35 kg
Rear tire mass (estimated)	35 kg
Front stiffness (Measured)	22,600 N/m
Rear stiffness (Measured)	46,800 N/m
Distance between front wheel and center of gravity (Measured)	1.3 m
Distance between rear wheel and center of gravity (Measured)	1.4 m

**Table 2:** The control parameters used to perform the experiments.

Controls	Parameters
*VFC comfort*	$g_{0}=-3.5\times 10^{4};\;g_{1}=0;\;g_{2}=2400;\;g_{3}=-1\times 10^{3}\;$
*VFC mix*	$g_{0}=-3.5\times 10^{4};\;g_{1}=-2\times 10^{5};\;g_{2}=1.5\times 10^{4};\;g_{3}=-5\times 10^{3}$
*VFC handling*	$g_{0}=-3.5\times 10^{4};\;g_{1}=0;\;g_{2}=1\times 10^{3};\;g_{3}=-600$
*Skyhook*	$g_{0}=0;\;g_{1}=0;\;g_{2}=40000;\;g_{3}=0$
